# Die infektiöse bakterielle Symphysitis (IBS; Symphysitis pubis purulenta) im urologischen Setting – eine deskriptive Studie und Einführung eines Therapiealgorithmus

**DOI:** 10.1007/s00120-025-02715-1

**Published:** 2025-10-30

**Authors:** F.-J. Dally, M. A. Rupp Pardos, D. Buergy, F. Giordano, N. Westhoff, S. Gravius, U. Obertacke, F. Bludau

**Affiliations:** 1 Orthopädisch-Unfallchirurgisches Zentrum (OUZ), Universitätsmedizin Mannheim (UMM), Mannheim, Deutschland; 2 Klinik für Strahlentherapie und Radioonkologie, Universitätsmedizin Mannheim (UMM), Mannheim, Deutschland; 3 Klinik für Urologie und Urochirurgie, Universitätsmedizin Mannheim (UMM), Mannheim, Deutschland; 4 Medizinische Fakultät Mannheim, Universität Heidelberg, Mannheim, Deutschland

**Keywords:** Symphysitis, Osteomyelitis pubis, Infektion des Beckenringes, Operative Symphysitisbehandlung, Osteitis pubis, Symphysitis, Osteomyelitis pubis, Infections of the pelvic ring, Operative therapy of a symphysitis, Osteitis pubis

## Abstract

**Einleitung:**

Eine infektiöse bakterielle Symphysitis (IBS) ist ein schwerwiegendes Krankheitsbild der Symphyse und der parasymphysären Strukturen. Nach einem operativen Eingriff, einer Intervention am unteren Harntrakt oder einer Bestrahlung im kleinen Becken kann sich eine IBS entwickeln.

**Ziel der Studie:**

Wir berichten über eine der größten urologischen Kohorten mit posttherapeutischer IBS, mit dem Ziel, die Aufmerksamkeit für dieses schwere Krankheitsbild zu erhöhen und die Diagnostik und Therapie darzustellen.

**Methode:**

Wir führten eine deskriptive, retrospektive Datenauswertung an einer Universitätsklinik über die Jahre 2018–2022 durch. 25 Patienten wurden wegen urologischer Grunderkrankungen behandelt und sind Bestand dieser Analyse.

**Resultate:**

Bei 25 Patienten mit einer IBS wurden als Grunderkrankung 24 Malignome nachgewiesen, 19/24 (79 %) Prostatakarzinome und 5/24 (21 %) Urothelkarzinome. Zwei Patienten wurden wegen nicht malignomassoziierten urologischen Erkrankungen behandelt. Das durchschnittliche Intervall bis zur Diagnose einer IBS betrug 73,9 (SD ± 55,7) Monate. In 14/25 (56 %) Fällen wurden Fistelformationen nachgewiesen, am häufigsten (8/14; 57 %) zwischen Blase und Symphyse. 11/25 (44 %) der Patienten konnten konservativ therapiert werden, 14/25 (56 %) mussten teilweise komplexen, interdisziplinären, rekonstruktiven und v. a. multiplen operativen Maßnahmen zugefügt werden.

**Schlussfolgerung:**

Eine IBS ist ein schweres Krankheitsbild, das im Rahmen und auch noch Jahre nach urologischen und uroonkologischen Behandlungen auftreten kann. Eine interdisziplinäre Therapie mit der Orthopädie/Unfallchirurgie sollte ebenso wie eine Versorgung in einem Zentrum mit entsprechender Erfahrung, Fallzahl und Infrastruktur stets angestrebt werden. Da die Diagnose und Therapie anspruchsvoll sind, präsentieren wir einen Algorithmus, der die Diagnostik beschreibt und anhand dessen die Therapie abgeleitet werden kann.

**Graphic abstract:**

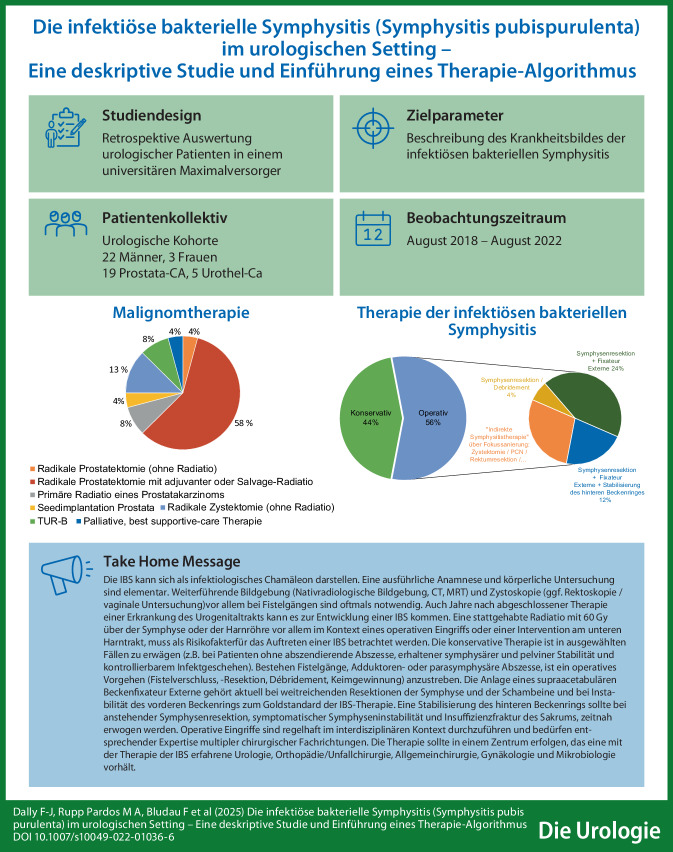

## Abstract (en)

**Background:**

Purulent infection of the pubic symphysis (infectious bacterial symphysitis, IBS) is a rare but serious disease of the symphysis and parasymphyseal structures. It can develop following surgery, interventions in the lower urinary tract, or pelvic radiation therapy.

**Objective:**

We report on one of the largest urological cohorts with post-therapeutic IBS, with the aim of increasing awareness for this severe condition and optimizing diagnosis and treatment.

**Methods:**

We conducted a descriptive retrospective data analysis at a university hospital over the years 2018–2022. Of the total collective, 25 patients were treated for underlying urological diseases and are part of this subgroup analysis.

**Results:**

Of the 25 patients with IBS, 24 were suffering from or had been treated for urological malignancies (19/24 [79%] prostate cancer; 5/24 [21%] urothelial cancer). Two patients had been treated for nonmalignant urological diseases. The average interval between surgery or intervention in the lower urinary tract due to the underlying urological disease and the diagnosis of IBS was 73.9 months (SD ± 55.7 months). Relevant fistula formations were detected in 14/25 (56%) cases, most frequently between bladder and symphysis (8/14, 57%). Of the 25 patients, 11 (58%) could be treated conservatively, while 14 (56%) had required complex, interdisciplinary, reconstructive, and, above all, multiple surgical procedures.

**Conclusion:**

Purulent infection of the pubic symphysis is a severe clinical condition that can occur during—and even years after—urological and uro-oncological treatments. This study illustrates the importance of ruling out IBS even years after urological disease or surgery or interventions in the lower urinary tract. Interdisciplinary treatment involving colleagues from orthopedic and trauma surgery should always be sought, as should treatment in a center with the appropriate experience, case numbers, and infrastructure. As diagnosis and treatment are demanding, creation of an interdisciplinary guideline could be useful. We present an algorithm which includes diagnostic steps and rational therapeutic options.

## Einleitung

Vorangestellt werden muss eine Klärung der Begrifflichkeit der Symphysitis: Der Begriff Symphysitis beschreibt eine Entzündung der Symphyse (Schambeinfuge). Im Begriff Symphysitis werden üblicherweise abakterielle und bakterielle Entzündungszustände der Symphyse und der umgebenden Gewebe (Knochen, z. B. Schambein [Os pubis] und Muskulatur [z. B. Adduktorenloge, Bauchmuskulatur und Faszien]) zusammengefasst. Wie bereits u. a. durch Fridrich et al. empfohlen, sollte aus Gründen der Verständlichkeit auf die begriffliche Zusammenfassung verzichtet werden [2]. Es wird empfohlen, klar eine infektiöse, bakterielle Symphysitis (IBS, „infectious inflammation of pubic symphysis“, Symphysitis pubis purulenta) von einer abakteriellen Symphysitis abzugrenzen [2]. Die abakterielle Symphysitis unterscheidet sich in Klinik, Genese, Diagnostik und Therapie sowie Morbidität und Mortalität entscheidend und stellt ein komplett unterschiedliches Krankheitsbild dar. Sie ist ein Krankheitsbild, an dem v. a. aktive Sportler und Schwangere erkranken und bedarf (außer im absoluten Ausnahmefall) keinerlei operativer Intervention. Die abakterielle Symphysitis war nicht Gegenstand dieser Studie und im Folgenden wird darauf nicht weiter eingegangen.

Die IBS wurde zuerst in einem Case Report von 1973 im Rahmen von hämatogen gestreuter Sepsis bei Patienten mit i.v.-Drogenabusus von Kido et al. erwähnt [5]. Hierbei wurde die radiologische Destruktion des Os pubis und der Symphyse in einer nativradiologischen Beckenübersichtsaufnahme beschrieben und der Keimnachweis in den Blutkulturen bei multifokalen septischen Arrosionen (Wirbelsäule, Schulter, Rippen, Symphyse) als beweisend für eine IBS gewertet [5].

Seit 1973 wurden Case Reports mit summativ 100 Fällen über die IBS publiziert [8]. Die IBS ist und bleibt damit eine seltene Erkrankung, welche destruierend sein und für den Patienten hoch dramatische Verläufe annehmen kann. Hansen et al. publizierten erst kürzlich eine etwa gleich große Kohorte, allerdings bestehend aus einem heterogenen Patientenkollektiv [3].

Interessanterweise stellen sich Patienten mit einer IBS im Nachgang einer urologischen Behandlung nicht standardmäßig bei ihrem behandelnden Urologen vor, sondern z. B. beim Gynäkologen, Allgemeinchirurgen, beim Orthopäden [1, 8–10] oder durchlaufen eine Odyssee an ärztlichen Vorstellungen und Untersuchungen bis die korrekte Diagnose gestellt wird [3]. Die Patienten beklagen Hüft- oder Leistenschmerzen, Schmerzen über der Symphyse beim Gehen und Stehen, beim Treppensteigen und beim Einbeinstand. Der charakteristische Druckschmerz über der Symphyse um das Os pubis ist ebenso vorhanden bei der abakteriellen Symphysitis und erschwert die Diagnose der IBS weiter. Klinisch präsentieren sich die Patienten in den überwiegenden Fällen mit höchstens mildem Fieber [10].

Biomechanisch sind im aufrechten Gang 20–30 % der Belastung über den vorderen Beckenring gerichtet, 70–80 % der Belastung beim aufrechten Stand und Gang verlaufen über das Sakrum, das Sakroiliakalgelenk, den hinteren Anteil des Beckenrings und schließlich in die unteren Extremitäten [7]. Wirkt die Erkrankung der Symphyse destabilisierend auf den vorderen Beckenring aufgrund von Infektion, Abszessen usw., so ist die Integrität des gesamten Beckenringes geschädigt. Folge dessen sind abnorme Belastungen und Scherkräfte des hinteren Beckenringes, die über die Zeit Insuffizienzfrakturen, ähnlich osteoporotischer Sakrumfrakturen bedingen können und mitunter hoch komplexe Operationen und Rekonstruktionen notwendig machen.

Unter Umständen kann eine konservative Therapie, bestehend aus Antibiotikatherapie ohne operative Intervention sinnvoll und erfolgversprechend sein. Diesbezüglich existiert allerdings keine hochgradige Evidenz oder eine Leitlinie, anhand derer Betroffenen eine dezidierte Empfehlung ausgesprochen werden könnte. Bei Abszessbildung und abszedierenden Verläufen, z. B. in die Adduktorenloge, das kleine Becken oder in den Oberschenkel hinein, oder Entstehung eines Fistelganges nach außen, ist ein operatives Débridement mit Drainage und ggf. Resektion des Fistelgangs zumeist indiziert. Sollte es zu einer destabilisierenden Destruktion der Symphyse mit Diastase über 2 cm [4] bzw. Instabilität kommen, so kann eine Stabilisierung der Symphyse mit interner Osteosynthese oder Fixateur externe sinnvoll sein. Die operative Therapie wird flankiert durch die Gabe von Antibiotika/Antimykotika, teilweise für 6–12 Wochen je nach Keimnachweis und Resistenzlage [3]. Gegebenenfalls ist nach Abklingen der Infektion eine Arthrodese oder interne Fixation der Symphyse mit einer Plattenosteosynthese erforderlich.

In dieser Studie beschreiben wir eine der größten IBS-Kohorten nach Operationen oder Interventionen am unteren Harntrakt und anschließender Bestrahlung im kleinen Becken.

## Methoden

Die Datenerhebung wurde über einen Zeitraum von 4 Jahren (August 2018–August 2022) durchgeführt. Es handelt sich um eine rein deskriptive Arbeit.

Wir analysierten alle diagnostizierten infektiösen, bakteriellen Symphysitiden in diesem Zeitraum. Identifiziert wurden die IBS über die Bilddatenbank der Radiologie. Wir führten eine Subgruppenanalyse mit dem Fokus auf IBS bei Patienten mit zugrunde liegenden urologischen Erkrankungen, Operationen oder Interventionen durch, da diese die Kohorte der IBS in unserer Auswertung anteilig, deutlich dominierten.

Die Diagnose IBS wurde zunächst durch einen Facharzt der Orthopädie/Unfallchirurgie über die gängige Definition bestätigt. Diese definiert sich über Schmerzen in der Schambeinregion, v. a. unter Belastung, radiologische Destruktion der Symphyse oder parasymphysären Strukturen oder Abszessformation in der Symphyse oder der parasymphysären Strukturen (Kontrastmittelaufnahme [KM] in der Computertomographie [CT]), Vorhandensein eines Fistelganges, der sich über der Symphyse entleerte, Auffinden einer putriden Destruktion intraoperativ oder Keimnachweis aus den intraoperativen Proben, analog zum Vorgehen von Hansen et al. [3].

Wir analysierten retrospektiv die Krankheitsverläufe, die bildgebenden Verfahren, die diagnostischen und therapeutischen Schritte und histologischen und mikrobiologischen Proben. Ebenso evaluierte ein Facharzt der Strahlentherapie die Bestrahlungspläne, wenn eine Radiatio zu einem Zeitpunkt der Behandlung stattgefunden hatte.

Mittels der radiologischen Software Syngo® (Siemens Healthineers AG, München, Deutschland) wurde eine Schlagwortsuche durchgeführt. Die gesuchten Schlagwörter waren „Symphysitis“, „Osteomyelitis pubis“ und „vesikosymphysäre Fistel“. Alle Treffer wurden anschließend weiterführend anhand der Patientenakten, durchgeführten Operationen/Punktionen/Interventionen, mikrobiologischen Befunde und therapeutischen Abläufe evaluiert und von einem Facharzt der Orthopädie validiert.

Konnten Patienten identifiziert werden, die eine postoperative oder postinterventionelle Radiatio des Tumorbettes, des OP-Gebiets oder des kleinen Beckens erhielten, wurde die Beteiligung der Symphyse hinsichtlich der applizierten Strahlendosis evaluiert.

Um Bestrahlungspläne der Patienten auszuwerten, wurde die MOSAIQ®-Software der Firma Elekta (Elekta, Stockholm, Schweden) genutzt. Mit Hilfe der Patientenarchivierungssoftware SAP/EPOS (SAP SE, Walldorf, Deutschland) wurden alle verfügbaren Informationen der betroffenen Patienten erneut eingesehen. Neben der Patientengeschichte wurde auch die durchgeführte Bildgebung (Magnetresonanztomographie [MRT] und CT) individuell mit der Software Syngo® (Siemens Healthineers AG, München, Deutschland) intermodal ausgewertet und auf das Vorhandensein einer Symphysitis geprüft. Die statistische Erfassung und Auswertung wurden mittels Microsoft Excel® (Microsoft Corp. Excel 2019, Washington, USA) durchgeführt.

Ein positives Ethikvotum wurde vor Beginn der Studie von der Ethikkommission II der Universität Heidelberg eingeholt – Zeichen 2021-896.

## Resultate

Männlich waren 22/25 (88 %) Patienten, 3/25 (12 %) waren weiblich.

Im Mittel waren die Patienten 79 (Range: 60–87) Jahre alt (Tab. 1). Es wurden 25 Symphysitiden im klinischen Kontext nach Therapien urologischer Erkrankungen diagnostiziert und behandelt, darunter 23/25 (92 %) aufgrund von Malignomen. 2/25 (4 %) Patienten wiesen kein Malignom auf. Eine IBS zeigte sich nach Therapie einer Schrumpfblase als Folge einer interstitiellen Zystitis, welche im Verlauf operativ mit Zystektomie und Anlage eines Ileozäkalpouches mit Appendixstoma therapiert wurde. Eine IBS entstand aufgrund einer vesikosymphysären Fistel nach Blasenverletzung durch ein Herniotomienetz. Die Mehrheit, 19/25 (76 %), wurde zuvor aufgrund von Karzinomen der Prostata, 5/25 (20 %) Patienten aufgrund von Urothelkarzinomen, behandelt.

**Tab. 1:** Charakteristiken der Studienpopulation

Parameter	*N* (%)
*Infektiöse bakterielle Symphysitis*	*N* *=* *25*
Medianes Erkrankungsalter	79,2 Jahre (Range 67–87 Jahre)
*Geschlecht*	
Männlich	22 (88)
Weiblich	3 (12)
*Medianes Zeitintervall zur Diagnose*	73,9 Monate (SD ± 55,7 Monate)
*Malignomnachweise*	*N* *=* *24*
*Anzahl Patienten mit Malignomen *(1 Patient wies im Verlauf zeitversetzt ein zweites urologisches Karzinom auf)	*N* *=* *23*
Prostatakarzinom	19 (79)
Urothelkarzinom	5 (21)
*Nicht Malignome*	*N* *=* *2*
Schrumpfblase nach interstitieller Zystitis	1 (50)
Blasenverletzung durch Herniotomienetz	1 (50)
Nicht-malignomassoziierte Zystektomien	2 (100)
*Malignomtherapie*	*N* *=* *24*
Radikale Prostatektomie (ohne Radiatio)	1 (4)
Radikale Prostatektomie mit adjuvanter oder Salvage-Radiatio	14 (58)
Primäre Radiatio eines Prostatakarzinoms	2 (8)
Seed-Implantation bei Prostatakarzinom	1 (4)
Radikale Zystektomie (ohne Radiatio)	3 (13)
Transurethrale Resektion der Harnblase	2 (8)
Palliative, Best-supportive-care-Therapie	1 (4)
*Therapie der infektiösen, bakteriellen Symphysitis*	*N* *=* *25*
Konservativ	11 (44)
Operativ	14 (56)
„Indirekte Symphysitistherapie“ über Fokussanierung: Zystektomie/PCN/Rektumresektion/Fistelsanierung/BDK	4 (17)
Symphysenresektion/Débridement	1 (4)
Symphysenresektion + Zystektomie + Fixateur externe	6 (25)
Symphysenresektion + Zystektomie + Fixateur externe + Stabilisierung des hinteren Beckenrings	3 (12)
*Fistelnachweis*	*N* *=* *14*
Vesikosymphysäre Fistel	8 (57)
Vesiko(symphysär)kutane Fistel	2 (14)
Prostatosymphysäre Fistel	1 (7)
Urethrosymphysäre Fistel	1 (7)
Urethroperineale Fistel	1 (7)
Urethrorektale Fistel	1 (7)

*TURB* transurethrale Resektion der Harnblase, *PCN* perkutane Nephrostomie, *BDK* Blasendauerkatheter

Im Rahmen der Therapie der malignen Grunderkrankung wurden 20 Patienten bestrahlt. Eine relevante Strahlendosis an der Symphyse und der Harnröhre war hier in der Hälfte der Fälle aufgrund einer nicht eruierbaren Bestrahlungsplanung nicht quantifizierbar. Diese Gebiete lagen nach Auswertung der Bestrahlungspläne allerdings im erweiterten Bestrahlungsfeld. Bei den quantifizierbaren 10 Patienten lag der Durchschnitt der an der Symphyse und Harnröhre applizierten Strahlendosis bei > 60 Gy.

**Abb. 1:**
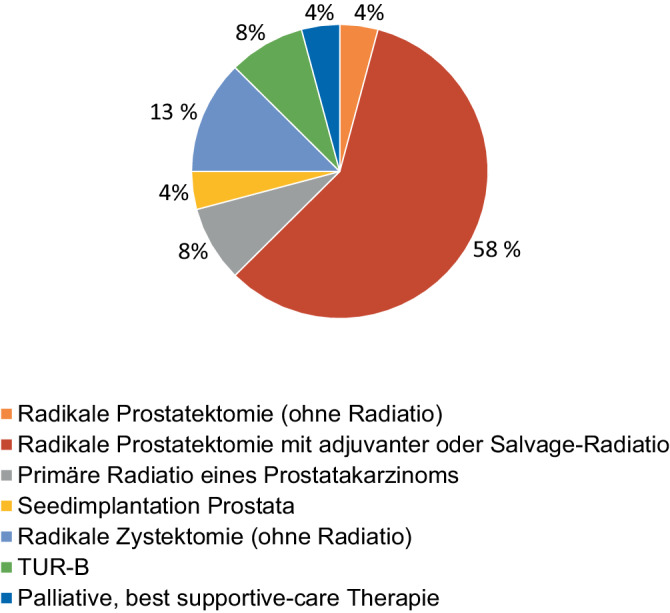
Malignomtherapie: Darstellung der Malignomtherapien für die 23 IBS-Patienten (infektiöse bakterielle Symphysitis) mit 24 Malignomnachweisen in der Vorgeschichte

Es wurden 24 Malignome bei 23 Patienten in der Kohorte nachgewiesen, in einem Fall wurde ein Urothelkarzinom 3 Jahre nach einem Prostatakarzinom diagnostiziert und ebenfalls therapiert (Zystektomie nach bereits erfolgter Prostatektomie drei Jahre zuvor). 19/23 (82 %) der Patienten wurden initial aufgrund eines Prostatakarzinoms therapiert, 5/23 (17 %) der Patienten wurden dementsprechend aufgrund eines Urothelkarzinoms therapiert. Bei einem Patienten erfolgte eine perkutane Seed-Implantation, bei 2 Patienten erfolgte eine transurethrale Resektion eines Urothelkarzinoms in der Harnblase (Abb. 1).

## Diagnose der IBS

Der größte Teil der Patienten (12/25, 48 %) stellte sich aus eigenem Anlass in der Notaufnahme oder in der orthopädischen Sprechstunde unseres universitären Maximalversorgers mit unspezifischen Schmerzen in der Leiste, im Unterbauch oder über der Symphyse vor. Die meisten der Patienten (20/25, 80 %) wiesen Symptome einer systemischen Infektion wie Fieber oder einen reduzierten Allgemeinzustand auf.

Der zweitgrößte Teil (8/25, 32 %) der Diagnosen wurde per Zufall im Rahmen von CT- oder MRT-Nachsorgeuntersuchungen (Strahlentherapie oder Urologie) gestellt. 5/25 (20 %) der Diagnosen wurden aufgrund der Verdachtsdiagnose vom niedergelassenen Hausarzt oder Urologen zur weiteren Abklärung vorgestellt.

Das durchschnittliche Intervall zwischen der Operation oder Intervention am unteren Harntrakt bis zur Diagnose einer IBS betrug 73,9 (SD ± 55,7) Monate.

Kardinalsymptome der Patienten mit IBS waren Druckschmerzen über der Symphyse, den Leisten, den Oberschenkeln oder im Unterbauch (insbesondere bei Belastung 10/25 [40 %]), chronische, als ausstrahlend imponierende, Leistenschmerzen 5/25 (20 %), schmerzbedingte Bewegungseinschränkungen 3/25 (12 %) und seltener Fieber 2/25 (8 %).

In 14 Fällen wurde eine Fistel nachgewiesen, welche die Diagnose IBS, übereinstimmend mit Keimnachweis und Bildgebung, bestätigte (Tab. 2). In 8 dieser 14 Fälle (57 %) zeigte sich dabei eine vesikosymphysäre Fistel. Je 1 Patient (7,1 %) zeigte eine prostatosymphysäre, eine urethrorektale Fistel, eine urethroperineale Fistel, eine urethrosymphysäre Fistel und eine prostatosymphysäre Fistel. Zwei Patienten (2/14; 14 %) zeigten eine vesikosymphysärekutane Fistel. Bei 8/14 (56,8 %) Patienten mit Fisteln fanden sich ebenfalls Abszesse im kleinen Becken oder bis in die Adduktorenloge reichend.

**Tab. 2:** Fistelnachweise

Fistelnachweis	*N* = 14
Vesikosymphysäre Fistel	8 (57)
Vesiko(symphysär)kutane Fistel	2 (14)
Prostatosymphysäre Fistel	1 (7)
Urethrosymphysäre Fistel	1 (7)
Urethroperineale Fistel	1 (7)
Urethrorektale Fistel	1 (7)

Die urethrorektale Fistel 1/14 (7 %) wurde per Rektosigmoidoskopie diagnostiziert und machte eine tiefe anteriore Rektumresektion notwendig.

In den meisten Fällen 10/14 (71 %) wurde der Verdacht eines symphysären Fistelgangs per CT gestellt. In allen Fällen wurde präoperativ im Rahmen der Diagnostik, eine Zystoskopie durchgeführt, jedoch war die Fistel nicht in allen Fällen zystoskopisch passier- oder darstellbar. Eine Bestätigung der Fistel gelang somit in 9/14 (64 %) Fällen. 13/14 (93 %) Patienten mit Fistelgängen litten ursprünglich an einem Prostatakarzinom. 1/14 (7 %) erlitt eine vesikosymphysäre Fistel aufgrund einer Blasenverletzung durch ein Herniotomienetz. 11/13 Prostatakarzinompatienten mit Fistelgang wurden mittels radikaler Prostatektomie und adjuvanter Radiatio therapiert. 1/13 Prostatakarzinompatienten mit Fistelgang und IBS wurde mittels palliativer TURP (transurethrale Prostataresektion) und anschließender Radiatio der Prostataloge therapiert. Ein weiterer Prostatakarzinompatient mit Fistelgang und IBS wurde nur extrakorporal bestrahlt und erlitt hiernach eine Strahlenzystitis und Proktitis mit konsekutiver urethrorektaler Fistel.

### Keimgewinnung

Die Keimgewinnung erfolgte immer prinzipiell über eine Urinkultur, zusätzlich, wenn gut durchführbar über eine Punktion der Symphyse oder der parasymphysären Abszessformationen. Die zur Diagnostik qualitativ hochwertigste Keimgewinnung erfolgt nach unserer Meinung allerdings intraoperativ aus tiefen Gewebeproben und wurde stets bei sanierungsbedürftigem Befund angestrebt und durchgeführt. Konnte kein Keim direkt an der Symphyse oder aus einem fistulierenden Kompartiment nachgewiesen werden, wurde empirisch mit 2‑facher antibiotischer Therapie zunächst intravenös stationär für mindestens 10 Tage mit Flucloxacillin und Ceftriaxon therapiert. Anschließend wurde per Os mit Amoxycillin/Clavulansäure für weitere 8–10 Wochen behandelt, sodass eine Gesamttherapiedauer von 10–12 Wochen resultierte. Sollte ein relevanter, pathogener Keim in entsprechender Konzentration ≥ 10 × 6^9/l z. B. im Urin nachgewiesen worden sein, so wurde dieser Keim entsprechend therapiert und die begonnene empirische antiinfektive Therapie keimgerecht umgestellt.

Wurde ein Patient mit Fistelgang therapiert, so überdauerte die antibiotische Therapie regelhaft 12 Wochen. In 11/25 (44 %) Fällen konnte kein Keim nachgewiesen werden. Es wurden in unserer Kohorte nur in Ausnahmefällen 2/14 (14 %) Monoinfektionen, dann stets mit *Staphylococcus aureus, *festgestellt. In allen anderen Fällen (12/14; 86 %) wurden Mischinfektionen nachgewiesen (Tab. 3). Die Therapie wurde stets mit der Mikrobiologie abgesprochen.

**Tab. 3:** Keimnachweise

Keimnachweis	*N* = 32
Staphylococcus spp.	6 (19)
Staphylococcus aureus	2 (6)
Staphylococcus epidermidis	3 (10)
Escherichia coli	6 (19)
Pseudomonas aeruginosa	2 (14)
Enterococcus faecalis	2 (6)
Enterococcus faecium	1 (3)
Bacteroides fragilis	2 (6)
Klebsiella pneumoniae	2 (6)
Streptococcus constellatus	1 (3)
Aerococcus urinae	1 (3)
Citrobacter freundii	1 (3)
Candida spp.	3 (10)

### Stabilität der Symphyse

Die Stabilität, respektive Instabilität der Symphyse wurde präoperativ über Schmerzen unter Belastung oder beim Einbeinstand (ins kleine Becken, Leiste oder in die Oberschenkel ziehend), bei radiologischer Diastase der Symphyse über 2 cm in der Becken Einbeinstandaufnahme (auch Flamingo-Aufnahme genannt) oder der stehenden konventionell-radiologischen Übersichtsaufnahme des Beckens diagnostiziert.

Bei allen Patienten mit relevanter Symphysenbeteiligung oder Instabilität wurde eine CT + KM durchgeführt. Im Rahmen der Krankenhausbehandlung erhielten allerdings alle Patienten in dieser Kohorte zu einem Zeitpunkt eine CT-Untersuchung. 11/25 (44 %) der Patienten erhielten ebenso eine MRT + KM-Untersuchung. Die MRT wurde nicht bei allen Patienten durchgeführt, da der Befund im nativen Röntgen mit erfolgter Zystoskopie und durchgeführter CT bereits ausreichend dargestellt war. Die MRT erfolgte regelhaft im Rahmen der Nachsorgeuntersuchungen und bei nicht komplett einschätzbaren Befunden an der Symphyse. Die CT und die MRT sind auch zur Einschätzung der Integrität des hinteren Beckenrings und dessen Stabilität wertvoll und sollten hier rasch erwogen werden.

Bei der Mehrheit der Patienten mit symptomatischer Symphyseninstabilität war der Einbeinstand regelhaft schmerzbedingt nicht möglich.

Jeder Patient mit einer relevanten Insuffizienzfraktur des hinteren Beckenrings war schmerzbedingt bettlägerig.

## Therapie

**Abb. 2:**
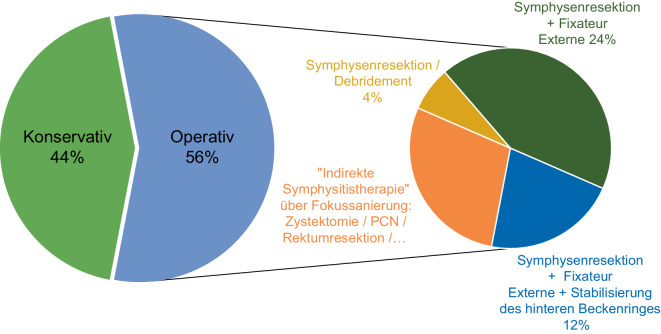
Therapie der infektiösen bakteriellen Symphysitis

Bei fehlender symphysärer Instabilität, Abwesenheit von Abszessen oder infauster Prognose der malignen Grunderkrankung, wurde bei 11 Patienten (11/25; 44 % – alle ohne Fistelnachweis) ein „Wait-and-see“-Ansatz bzw. ein konservativer Therapieansatz mit oder ohne antiinfektiver Therapie nach Diagnose der IBS gewählt (Abb. 2).

Bei 7 dieser Patienten (63 %) wurde die IBS als „okkulte“ Diagnose zufällig im Rahmen von Schnittbildgebungen zur Nachsorge der Grunderkrankung gestellt. Hier hatten sich, weder Infektzeichen noch Schmerzen oder andere behandlungsbedürftige Symptome einer IBS gezeigt. Im Konsens mit den Patienten wurden diese Patienten auch ohne Antibiotikatherapie einer Surveillance zugeführt. In den Follow-up-Untersuchungen zeigte sich keine Zunahme der bildmorphologischen IBS. Das MRT mit KM war hier die Untersuchungsmodalität der Wahl.

Bei 2/11 (18 %) der Patienten wurde nach Keimgewinnung per Symphysenpunktion eine keimgerechte antiinfektive Therapie für 12 Wochen zugeführt. Die IBS heilte hier aus. Diese Patienten konnten ohne Bedarf einer operativen Therapie entlassen werden und wurden im Beobachtungszeitraum auch nicht mehr auffällig.

Aufgrund ihres Alters und Vorerkrankungen waren 2/11 (18 %) der Patienten für eine operative Therapie nicht geeignet. Sie wurden einer Basistherapie zugeführt und auf Wunsch der Patienten nach Hause entlassen.

## Operative Therapie der IBS

Aufgrund der ausgeprägten, destruierenden Symphysitis mit Abszedierungen und Fistelformationen wurden 10/25 (40 %) Patienten einer interprofessionellen (urologisch-orthopädisch) operativen Therapie bestehend aus Symphysenresektion, Fistelresektion, Abszessausräumung, Débridement, Nekrosektomie, ggf. Anlage eines supraacetabulären Fixateur externe und in 3 Fällen Stabilisierung des hinteren Beckenrings, flankiert von antiinfektiver Therapie, zugeführt.

Die Fisteltherapie richtete sich nach der anatomischen Lokalisation und „Verbindung“. Neben Fistelexzision wurde regelhaft (10/10) eine Zystektomie notwendig. Ebenso wurden mitunter weitere Fachkliniken im Verlauf in die operative Therapie mit einbezogen (Allgemeinchirurgie und Gynäkologie).

Die perioperative Antibiotikatherapie wurde nach dem oben genannten internen Standard oder entsprechend im Urin nachgewiesener Keime festgelegt und nach Keimidentifikation entsprechend antibiogrammgerecht oder antimykotisch umgestellt. Nach operativer Sanierung des symphysären Infektfokus wurde die Antibiotikatherapie auf 6 Wochen limitiert. Diese Dauer wurde in den meisten Fällen aufgrund weiterer operativer Maßnahmen und weiterer Keimnachweise regelhaft überschritten und notwendigerweise ausgedehnt.

Bei vorliegender Symphysenstabilität (4/24 [16 %]) konnte zunächst kausal die Fistelsanierung ohne orthopädisch-operative Maßnahme vorgenommen werden (Abb. 2) Die IBS heilte anschließend durch antiinfektive Therapie konservativ ohne direkte operative Maßnahme an der Symphyse aus. Interessanterweise heilte bei 2/4 (50 %) die IBS nach Trockenlegung der vesikosymphysären Fistelgänge per PCN (perkutane Nephrostomie) und somit ohne operative Fistelresektion aus. Je 1/4 (25 %) Patienten wurde kausal zur Fistelbeseitigung per Zystektomie oder Rektumresektion mit entsprechend keimgerechter antiinfektiver Medikation therapiert. Auch hier heilte die IBS ohne weitere notwendige Intervention oder operative Maßnahme an der Symphyse aus.

Bei Instabilität der Symphyse oder Notwendigkeit der weitreichenden Symphysen- und Os-pubis-Resektion erfolgte die temporäre Anlage eines Fixateur externe des Beckenrings (9/10; 90 %) im Durchschnitt für 60 (SD ± 21) Tage. Die Anlagen erfolgten bei allen Patienten operativ als supraacetabulärer Fixateur externe, die Entfernungen erfolgten zumeist unter Lokalanästhesie. 1/10 (4 %) erhielt nur ein Symphysendébridement, da die Symphyse intraoperativ als stabil bewertet wurde.

Im Rahmen einer Zystektomie und Sanierung der Symphysitis bei vorhandenem Fistelgang wurde stets im gleichen urologisch-orthopädischen interdisziplinären Eingriff auch der Fixateur externe angelegt.

Es wurde stets intraoperativ mindestens eine histopathologische Probe entnommen und untersucht. Die IBS wurde somit, klinisch, bildmorphologisch, mikrobiologisch und histopathologisch nachgewiesen. In den Fällen der operativen Symphysenresektion oder des Symphysendébridements wurde die Diagnose per florider Osteomyelitis oder passenden Fistelganganteilen histopathologisch bestätigt.

Eine Insuffizenzfraktur des hinteren Beckenrings erlitten 3/10 (12 %) Patienten mit stattgehabter Symphysenresektion oder Instabilität. In diesen Fällen wurde der hintere Beckenring mittels ISG (Iliosakralgelenk)-Verschraubung, ISG-Fusionsimplantat oder lumbopelviner Stabilisierung versorgt. Nach durchgeführter additiver Stabilisierung des hinteren Beckenringes besserte sich die Mobilität dieser Patienten entscheidend.

Die Mortalität lag in unserer Kohorte bei 5/25 (20 %). Die Patienten, die verstarben, sind direkt oder indirekt an den Folgen der IBS verstorben. 3 Patienten verstarben aufgrund eines schweren septischen Verlaufs, 1 Patient aufgrund postoperativer Komplikationen. Eine Patientin verstarb unabhängig von der IBS während des stationären Aufenthalts an ihrer uroonkologischen Grunderkrankung.

Der Behandlungserfolg wurde am Rückgang der labormedizinisch gemessenen infektiologischen Laborwerte, Beseitigung/Verschluss der Fistelgänge, Wiedererlangen der Mobilität der Patienten und Besserung der Schmerzen gemessen.

## Diskussion

Die IBS ist ein schweres Krankheitsbild, welches auch noch Jahre nach einer Therapie einer Erkrankung des Urogenitaltrakts auftreten kann.

Die Relevanz dieser Erkrankung liegt in ihrer hohen Morbidität und Mortalität, einhergehend mit der großen Latenz zur auslösenden Grunderkrankung, schwierigen Diagnose und mitunter hoch komplexen und langwierigen Therapie, die v. a. in der belastungsabhängigen Schmerzhaftigkeit der Symphyse, des hinteren Beckenrings, der zugrunde liegenden Therapie der Fistelgänge und der möglichen Ausbreitung der parasymphysären Abszesse begründet ist.

**Abb. 3:**
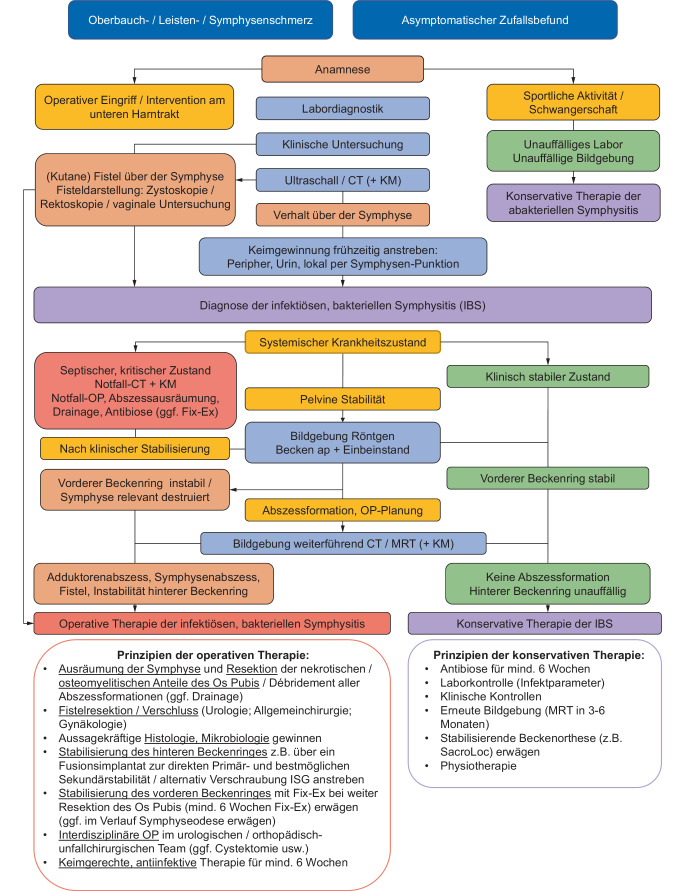
Diagnostischer und therapeutischer Algorithmus der Symphysitis (*CT* Computertomographie, *KM* Kontrastmittel, *MRT* Magnetresonanztomographie, *Fix-Ex* Fixateur externe, *ISG* Iliosakralgelenk *IBS* infektiöse bakterielle Symphysitis)

Um die notwendigen diagnostischen und therapeutischen Schritte bestenfalls abzubilden, empfehlen wir das strukturierte Vorgehen z. B. anhand des hier vorgestellten Algorithmus (Abb. 3).

Wir konnten in dieser retrospektiven Datenerhebung zeigen, dass die IBS höchst unspezifische Symptome auslöst, hierdurch regelhaft die Diagnose erschwert ist und in einem relevanten Anteil auch als Zufallsbefund im Rahmen von Nachsorgeuntersuchungen als klinisch okkulte Situation imponieren kann.

Zuvor bestrahlt wurden 20 Patienten (80 %). Nur bei 10 dieser Patienten konnte eine relevante Strahlendosis für die Symphyse oder die Urethra bestätigt werden. Die stattgehabte Radiatio im kleinen Becken mit Einbezug der Symphyse ist allerdings sicherlich als Kofaktor für die Entwicklung einer IBS zu betrachten. Insbesondere eine postoperative Minderperfusion der Symphyse muss hier allerdings ebenso als ursächlich in Betracht gezogen werden [6]. Mit Ausnahme eines Patienten, dessen vesikosymphysäre Fistel auf dem Boden einer Blasenverletzung durch einen Fremdkörper (Herniotomienetz) herrührte, wurden alle Patienten mit einem Fistelgang im kleinen Becken im Rahmen ihrer vorangegangen Malignomtherapie bestrahlt. Eine Radiatio im kleinen Becken muss sicherlich ebenfalls wie der operative Eingriff im kleinen Becken als Risikofaktor für die Entstehung einer Fistel und somit, wie bereits erwähnt, einer IBS angesehen werden. Die Zunahme der Komplexität der Wundheilungsstörung oder der Fistulierung nach stattgehabter Radiatio deckt sich zumindest teilweise mit der Erfahrung, die Hansen et al. in ihrer Publikation beschreiben [3].

Eine relevante Anzahl unserer Studienkohorte präsentierte sich symptomarm oder symptomfrei und wurde im Rahmen von Nachsorgeuntersuchungen diagnostiziert. Hier ist das MRT mit KM die Untersuchungsmodalität der Wahl [3].

Unsere Auswertung zeigt, dass bei unspezifischen Symptomen und Schmerzen in der Schambein- oder Leistenregion und unklaren Infektwerterhöhungen an eine IBS gedacht werden muss. Dies unterstreicht die Wichtigkeit der ausführlichen Anamnese, körperlichen Untersuchung und interdisziplinären Zusammenarbeit zwischen Urologie, Strahlentherapie und Orthopädie.

Wie Ross et al. beschrieben, konnten wir in unserer Kohorte ebenso bestätigen, dass der Symphysen‑/Unterbauch- und Leistenschmerz zu den häufigsten Symptomen gehört. Allerdings lag in unserer Auswertung Fieber deutlich seltener vor (8 % vs. 74 % bei Ross et al. [8]), obwohl teilweise bereits ausgeprägte entzündliche Veränderungen nachweisbar waren.

Die Kohorte, die Ross et al. untersuchten, beschreibt 17 „pelvic malignancies“ als zugrunde liegende Grunderkrankungen, die die IBS bedingten. Unsere Kohorte setzt sich hier über die klare Distinktion hinsichtlich uroonkologischer Malignome ab, da Ross et al. hier nicht weiter differenzieren [8]. Darüber hinaus ist unsere Kohorte aus 25 uroonkologischen Patienten die größte bisher beschriebene Kohorte von IBS nach Operationen oder Interventionen am unteren Harntrakt.

Ähnlich zur Kohorte von Ross et al. konnten wir die polymikrobielle Besiedlung von IBS auf dem Boden von Malignomen bestätigen [8].

Anders als in der Kohorte von Hansen et al. wurde in unserer Kohorte nicht jeder Fall einer bakteriellen Symphysitis operativ versorgt [3]. Dies ist u. a. darauf zurückzuführen, dass einige der Patienten ein gutes Ansprechen auf die Antibiotikatherapie klinisch und laborchemisch zeigten und ein relevanter Anteil als okkulte IBS im Rahmen von Nachsorgeuntersuchungen imponierte.

Ebenso stellt sich heraus, dass die Therapie bei IBS mit sicherem Keimnachweis komplex ist und mitunter große, komplexe und multiple Operationen nach sich zieht, welche im interdisziplinären Setting durchgeführt werden müssen. Auch waren in unserer Kohorte zur Infektbehandlung und Fistelentfernung regelhaft mehrere operative Sitzungen und langwierige Antibiotikabehandlungen notwendig, was sich mit den Erkenntnissen von Ross et al. deckt [8].

Unsere Auswertung zeigt, dass die Biomechanik des Beckenrings relevant ist und eine sinnvolle Therapie die Anlage eines Fixateur externe des Beckens sein kann. Die notwendige Dauer der Anlage kann auch durch unsere Studie letztlich nicht geklärt werden, sicherlich ist aber eine Anlage über die Dauer unseres Mittelwerts von 60 Tagen zu empfehlen. Ebenso zu empfehlen ist anhand dieser Studie bei weitreichender Resektion der Schambeine oder großer Instabilität des vorderen Beckenrings, direkt an die Versorgung und Stabilisierung des hinteren Beckenrings zu denken. Wenngleich dies nur in 30 % der Symphysenresektionen notwendig wurde, so sind die Folgen für den einzelnen Patienten durchgreifend. Jeder Patient mit einer IBS-bedingten Insuffizienzfraktur des hinteren Beckenrings war schmerzbedingt bettlägerig. Allein schon zur Vermeidung von relevanten internistischen Erkrankungen und dem unabwendbaren Siechtum dieser schmerzgeplagten Patienten gebührt, die Stabilisierung des hinteren Beckenrings zeitnah durchzuführen. Wir empfehlen allen Patienten, die eine IBS-bedingte Symphysen‑/Schambeinresektion in Aussicht haben oder eine symptomatische Symphyseninstabilität aufweisen, additiv eine Stabilisierung des hinteren Beckenrings durchführen zu lassen.

Eine interdisziplinäre Behandlung der IBS-Patienten ist stets anzustreben. Die Behandlung von IBS-Patienten, v. a. derer mit vorliegenden Fistelgängen, gehört einem Zentrum der Maximalversorgung vorbehalten, das mindestens eine urologische, orthopädische/unfallchirurgische, allgemeinchirurgische, gynäkologische und mikrobiologische Abteilung vorweist.

In unserer Auswertung konnten wir zeigen, dass die konservative Therapie bei limitiertem Befall, bzw. im Frühstadium ebenfalls eine mögliche Therapieoption darstellt.

### Limitationen

Die vorliegende Studie ist retrospektiv erhoben worden, somit ist ein kausaler Zusammenhang zwischen Operationen/Interventionen des unteren Harntraktes sowie der einhergehenden Bestrahlung im kleinen Becken und dem Entstehen einer IBS nur annehmbar. Die Datenlage lässt einen deutlichen Zusammenhang vermuten.

## Schlussfolgerung

Auch noch Jahre nach einem operativen Eingriff, einer Intervention am unteren Harntrakt oder einer Bestrahlung im kleinen Becken kann sich eine IBS entwickeln. Der Zeitraum bis zur Diagnosefindung ist hierbei teilweise prolongiert, diffizil, mitunter zufällig und häufig verzögert. Es existiert keine einheitliche Behandlungsempfehlung zur Diagnostik oder Therapie der IBS. Wir konnten zeigen, dass die konservative Therapie eine valide Methode ist, um eine IBS zu therapieren. Wir konnten ebenso zeigen, dass eine postoperative Radiatio bzw. eine Radiatio des kleinen Beckens ein relevanter Risikofaktor für die Entstehung einer Fistel zwischen Symphyse und der Strukturen im kleinen Becken sein könnte.

Wir empfehlen ferner, wie bereits in der Literatur gefordert, den Begriff Symphysitis nicht mehr alleinstehend zu nutzen, sondern stets die eindeutigen Zusätze „infektiös, bakteriell“ anzuwenden, sollte es sich um eine septische Symphysitis handeln. Gleichfalls empfehlen wir stets den Zusatz „abakterielle Symphysitis“ oder „abakterielle Osteitis pubis“ anzuwenden, sollte es sich um die nicht-infektiöse Schambeinentzündung des Sportlers oder der Schwangeren handeln.

Bis zum heutigen Tag existiert keine Leitlinie oder Behandlungsempfehlung für Patienten mit einer IBS. Es gilt eine IBS auch noch Jahre nach einer urologischen Erkrankung, Operationen oder Interventionen am unteren Harntrakt auszuschließen. Dies v. a. wenn eine unspezifische Infektion vorzuherrschen scheint. Eine interdisziplinäre Therapie mit orthopädisch-unfallchirurgischen Kollegen (und weiteren Fachrichtungen) sollte ebenso wie eine Versorgung in einem Zentrum mit entsprechender Erfahrung, Fallzahl und Infrastruktur stets angestrebt werden. Da die Diagnose und Therapie anspruchsvoll sind, empfehlen wir die Nutzung des diagnostischen und therapeutischen Algorithmus aus Abb. 3.

## Fazit für die Praxis

- Die infektiöse bakterielle Symphysitis (IBS) kann als infektiologisches Chamäleon auftreten.
- Eine ausführliche Anamnese und körperliche Untersuchung sind elementar.
- Weiterführende Schnittbildgebung und Zystoskopie (ggf. Rektoskopie/vaginale Untersuchung) v a. bei Fistelgängen sind oft notwendig.
- Auch Jahre nach abgeschlossener Therapie einer Erkrankung des Urogenitaltrakts kann es zu einer IBS kommen.
- Eine Radiatio mit 60 Gy über der Symphyse oder der Harnröhre nach Eingriff oder Intervention am unteren Harntrakt, muss als Risikofaktor für eine IBS betrachtet werden.
- Die konservative Therapie ist in ausgewählten Fällen zu erwägen.
- Bestehen Fistelgänge, Adduktoren- oder parasymphysäre Abszesse, ist ein operatives Vorgehen (Fistelverschluss, -resektion, Débridement, Keimgewinnung) anzustreben.
- Die Anlage eines supraacetabulären Beckenfixateur externe gehört aktuell bei weitreichenden Resektionen der Symphyse und Instabilität des vorderen Beckenrings zum Goldstandard der IBS-Therapie.
- Eine Stabilisierung des hinteren Beckenrings sollte bei anstehender Symphysenresektion, symptomatischer Symphyseninstabilität und Insuffizienzfraktur des Sakrums angestrebt werden.
- Operative Eingriffe sind im interdisziplinären Kontext durchzuführen.
- Die Therapie sollte in einem Zentrum erfolgen, das eine geeignete Urologie, Orthopädie/Unfallchirurgie, Allgemeinchirurgie, Gynäkologie und Mikrobiologie vorhält.

## Data Availability

Die zugrunde liegenden Daten können mittels Anfrage beim Autor eingesehen/angefordert werden.
